# Impact of maternal depression on malnutrition treatment outcomes in older children with sickle cell anemia

**DOI:** 10.1186/s40795-024-00826-0

**Published:** 2024-01-24

**Authors:** Claire Ritter, Shehu U. Abdullahi, Safiya Gambo, Hassan Adam Murtala, Halima Kabir, Khadija A. Shamsu, Garba Gwarzo, Yasmin Banaei, Sari A. Acra, Virginia A. Stallings, Mark Rodeghier, Michael R. DeBaun, Lauren J. Klein

**Affiliations:** 1 Meharry Medical College, Nashville, TN USA; 2 Department of Pediatrics, Bayero University/Aminu Kano Teaching Hospital, Kano, Nigeria; 3 Department of Pediatrics, Murtala Mohammed Specialist Hospital, Kano, Nigeria; 4 Yasmin Banaei MD LLC, Washington, DC USA; 5 Department of Pediatrics, D. Brent Polk Division of Pediatric Gastroenterology, Hepatology, and Nutrition, Monroe Carell Jr. Children’s Hospital at Vanderbilt, Nashville, TN USA; 6 Department of Pediatrics, Division of Gastroenterology, Hepatology, and Nutrition, The Children’s Hospital of Philadelphia and University of Pennsylvania, Philadelphia, PA USA; 7 Rodeghier Consultants, Chicago, IL USA; 8 Department of Pediatrics, Vanderbilt-Meharry Center of Excellence in Sickle Cell Disease, Vanderbilt University Medical Center, Nashville, TN USA; 9 Vanderbilt Institute for Global Health, Nashville, TN USA

**Keywords:** Maternal depression, Sickle cell anemia, Malnutrition, Maternal health, Patient health questionnaire, Body mass index

## Abstract

**Background:**

Malnutrition and sickle cell anemia (SCA) result in high childhood mortality rates. Although maternal depression is an established risk factor for malnutrition in younger children, little is known about its impact on treatment response in children with malnutrition. We aimed to determine the relationship, if any, between maternal depression scores and malnutrition treatment outcomes in older children with SCA.

**Methods:**

We conducted a planned ancillary study to our randomized controlled feasibility trial for managing severe acute malnutrition in children aged 5–12 with SCA in northern Nigeria (NCT03634488). Mothers of participants completed a depression screen using the Patient Health Questionnaire (PHQ-9).We used a multivariable linear regression model to describe the relationship between the baseline maternal PHQ-9 score and the trial participant’s final body mass index (BMI) z-score.

**Results:**

Out of 108 mother-child dyads, 101 with maternal baseline PHQ-9 scores were eligible for inclusion in this analysis. At baseline, 25.7% of mothers (26 of 101) screened positive for at least mild depression (PHQ-9 score of 5 or above). The baseline maternal PHQ-9 score was negatively associated with the child’s BMI z-score after 12 weeks of malnutrition treatment (β=-0.045, *p* = 0.041).

**Conclusions:**

Maternal depressive symptoms has an impact on malnutrition treatment outcomes. Treatment of malnutrition in older children with sickle cell anemia should include screening for maternal depression and, if indicated, appropriate maternal referral for depression evaluation and treatment.

**Trial Registration:**

The trial was registered at clinicaltrials.gov (#NCT03634488) on January 30, 2018, https://clinicaltrials.gov/study/NCT03634488.

## Background

Malnutrition is an ongoing global public health concern, with sub-Saharan Africa accounting for 33% of all malnourished children globally [1]. Children with sickle cell anemia (SCA) are at an increased risk of malnutrition due to their high protein turnover rate, resulting in higher daily nutrient requirements [2–4]. Sub-Saharan Africa is home to over 75% of all newborns with SCA, with Nigeria accounting for over 30% of children born with SCA worldwide [5]. Children with SCA and malnutrition are at a higher risk for hospitalization [6] and mortality [7]. Addressing the double burden of malnutrition and SCA is crucial for decreasing childhood mortality, especially in sub-Saharan Africa, where the majority of affected children reside.

Maternal mental health, particularly depression, is an established risk factor for childhood malnutrition during the antenatal and postpartum periods [8–13]. Maternal depression is critical not only in the peripartum period but also as the child grows into adolescence and young adulthood. Maternal depression significantly impacts the health of older children and is commonly associated with reduced healthcare seeking [14], increased childhood illnesses [15], and poorer child diet quality [16]. Strong evidence indicates parents of children with SCA have a higher prevalence of depressive symptoms [17, 18], and mothers, in particular, are more likely to experience a lower quality of life [19]. 

Building on the prior evidence of the influence of maternal depression on the development of malnutrition, we tested the hypothesis that participants in a malnutrition trial whose mothers score higher on the depression screening tool, the Patient Health Questionnaire-9 (PHQ-9) [20], will experience less improvement in their body mass index (BMI) z-scores following treatment for malnutrition.

## Methods

### Study design

The planned ancillary study was completed as a prospective cohort study within a randomized controlled feasibility trial for managing severe acute malnutrition in children with SCA conducted in Nigeria (SAMS trial, NCT03634488) [21]. The SAMS trial included participants aged 5–12 years old with laboratory-confirmed SCA (HbSS or HbS-beta^0^ thalassemia) and uncomplicated severe acute malnutrition, as determined by a BMI z-score <-3.0 according to the World Health Organization growth reference [22]. Participants were recruited from Aminu Kano Teaching Hospital and Murtala Mohammed Specialist Hospital in Kano, Nigeria. Participants were randomly allocated to receive either age-based supplemental ready-to-use therapeutic food (RUTF; 500-1,000 daily calories) alone or a combination of RUTF and moderate fixed-dose hydroxyurea (20 mg/kg per day) for a 12-week treatment period. A total of 108 participants completed the trial. The main findings from the SAMS trial have been previously published [21]. This planned ancillary study and the main SAMS trial adhere to CONSORT reporting guidelines [23]. 

The Institutional Review Boards at Aminu Kano Teaching Hospital, Murtala Mohammed Specialist Hospital, and Vanderbilt University Medical Center approved the SAMS trial. All participants’ legal guardians provided written informed consent before screening and enrollment, and children over seven years of age provided assent. The study activities were carried out in accordance with the principles of the Declaration of Helsinki.

### Study sample

Within the cohort of participants who completed the SAMS trial (*n* = 108), our analysis focused on mother-child dyads where the mothers underwent baseline depression screening utilizing the PHQ-9. The exclusion criteria were primary caregivers who were not mothers, mothers of participants who did not complete the 12-week trial, and mothers who did not complete a PHQ-9 assessment at their initial visit. We decided to limit the PHQ-9 score analysis to only mothers to assess the impact of maternal depression on malnutrition treatment. This decision was based on the current body of evidence, which predominantly examines the correlation between maternal depression and nutritional status [8–10]. 

### Data collection

During the 12-week SAMS trial, participants presented to the clinic once for screening, followed by a visit to evaluate for refeeding within 5 days of study initiation, then every four weeks. At the initial visit, baseline medical history, demographics, physical examinations, and anthropometric measurements were conducted. Subsequently, at each follow-up visit, interim illness history, physical examinations, and anthropometric measurements were systematically performed per the study protocol [21]. Head of household education was used as a proxy for socioeconomic status.

In addition, at study enrollment and every subsequent clinic visit, the mother of the trial participant was assessed for depression using the PHQ-9, a publicly available depression screening tool [20, 24]. Each of the 9 criteria for depression per the Diagnostic and Statistical Manual of Mental Disorders: DSM-5 is evaluated on a scale of 0 (not at all) to 3 (nearly every day) [20]. The PHQ-9 can be scored as either a continuous variable, ranging from 0 to 27, where higher scores represent more severe depression, or can be interpreted categorically [24]. The PHQ-9 scores are categorized based on the severity of the depressive symptoms and are organized as follows: minimal (score of 0–4), mild (score of 5–9), moderate (score of 10–14), moderately severe (score of 15–19) or severe (score of 20–27) [20]. Additionally, the PHQ-9 has previously been validated to accurately identify individuals with depression in Nigeria [25]. Research staff were trained in the pre-trial period to administer the PHQ-9 verbally in Hausa, the most common local language. Translated versions of PHQ-9 have been previously validated in sub-Saharan African communities with consistent psychometric properties [26, 27]. The PHQ-9 yields similar results when administered verbally compared to when self-administered [28]. Mothers were referred to a local psychiatrist if their PHQ-9 score reached 9 or above during any clinic visit throughout the SAMS trial, as per the study protocol and prearranged agreement between the study team and the psychiatrist.

### Data analysis

Data were collected and managed with Research Electronic Data Capture (REDCap) hosted at Vanderbilt University Medical Center [29, 30]. We summarized continuous variables as means and standard deviations or as medians and interquartile ranges for variables not normally distributed. Categorical variables and prevalence were reported as counts and percentages. For comparisons between groups, a chi-square test or Fisher’s exact test was used for percentages, a t-test for means, and a Mann-Whitney U test for medians. A Pearson correlation was used to assess the relationship between baseline PHQ-9 scores and change in BMI z-scores. We used a linear regression model with covariates in the final model from the SAMS trial to study whether PHQ-9 scores at baseline were associated with the final BMI z-score. We used a two-sided P value of < 0.05 as evidence of a significant result for our a priori hypotheses. SPSS version 29.0.1 (IBM, Armonk, NY) was used for analysis.

## Results

### Baseline characteristics and depression scores for mother-child dyads

Among the 108 participants who completed the SAMS trial, 93.5% (101 mother-child dyads) met inclusion criteria, with exclusions based on missing baseline maternal PHQ-9 scores (*n* = 4) and the primary caregiver not being the mother (*n* = 3). Almost all mothers with known marital status (93.8%, *n* = 91 of 97) were married, with a median of 6 (IQR: 4.0–8.0) children in the household. The median PHQ-9 score at baseline for the whole cohort was 3 (IQR: 1–5). At baseline, 24.8% of mothers (*n* = 25) screened positive for mild depression (score of 5–9), and 1.0% of mothers (*n* = 1) screened positive for moderate depression (score of 10–15). Otherwise, there were no significant differences in baseline demographic and clinical information between mother-child dyads by whether mothers had a baseline PHQ-9 score of 5 or above (Table 1).

**Table 1:** Baseline characteristics of mothers and children, stratified by maternal baseline PHQ-9 score

Variables	Total cohort (*n* = 101)	Maternal Baseline PHQ-9 score < 5 (*n* = 75)	Maternal Baseline PHQ-9 score ≥ 5 (*n* = 26)	P value
Married, n (%), (*n* = 97)	91 (93.8)	66 (91.7)	25 (100.0)	0.334‡
Number of children in household, median (IQR)	6.0 (4.0–8.0)	6.0 (4.0–8.0)	6.5 (4.8–11.0)	0.230#
Total number of people in the household, median (IQR)	9.0 (6.5–12.0)	9.0 (6.0–11.0)	9.5 (6.8–16.2)	0.366#
Head of household education, n (%), (*n* = 100)				0.084‡
None/Primary/Jr. Secondary	33 (33.0)	20 (27.0)	13 (50.0)	
Sr. Secondary/OND	56 (56.0)	46 (62.2)	10 (38.5)	
University/Professional	11 (11.0)	8 (10.8)	3 (11.5)	
Child sex, female, n (%)	50 (49.5)	37 (49.3)	13 (50.0)	0.953*
Child age, yrs., median (IQR)	10.4 (8.8–11.6)	10.3 (8.4–11.5)	10.4 (9.6–12.2)	0.160#
Group, n (%)				0.953*
RUTF	50 (49.5)	37 (49.3)	13 (50.0)	
RUTF + hydroxyurea	51 (50.5)	38 (50.7)	13 (50.0)	
Child hemoglobin, g/dL, mean, (std. dev.)	7.4 (1.1)	7.3 (1.0)	7.6 (1.1)	0.224§

# Mann-Whitney test

‡ Fisher’s exact test

* Chi square test

§ T test

PHQ-9: Patient Health Questionnaire 9

RUTF: Ready-to-use therapeutic food

OND: Ordinary National Diploma

*There was no significant association between baseline maternal depression score and baseline child anthropometrics.*

All children were severely wasted per study criteria with a mean baseline BMI z-score of -3.7. There was also no significant difference between baseline BMI, weight-for-age, and height-for-age z-scores between women with or without at least mild baseline depression scores (PHQ-9 score of 5 or above; Table 2).

**Table 2:** Baseline and endpoint anthropometric z-scores of participants by maternal baseline PHQ- 9 score

Variables	Total cohort (*n* = 101)	Maternal Baseline PHQ-9 score < 5 (*n* = 75)	Maternal Baseline PHQ-9 score ≥ 5 (*n* = 26)	P value#
BMI z-score at baseline, mean (std. dev.)	-3.7 (0.5)	-3.7 (0.5)	-3.7 (0.5)	0.958
BMI z-score at 12 weeks, mean (std. dev.)	-3.2 (0.6)	-3.1 (0.6)	-3.3 (0.6)	0.296
Weight-for-age z-score at baseline, mean (std. dev.)	-3.6 (0.7)	-3.6 (0.6)	-3.4 (0.8)	0.312
Weight-for-age z-score at 12 weeks, mean (std. dev.)	-3.3 (0.7)	-3.3 (0.7)	-3.2 (0.8)	0.681
Height-for-age z-score at baseline, mean (std. dev.)	-2.3 (1.0)	-2.3 (0.9)	-2.1 (1.1)	0.312
Height-for-age z-score at 12 weeks, mean (std. dev.)	-2.3 (1.0)	-2.3 (1.0)	-2.1 (1.1)	0.412

# Chi-square test for counts; Two sample t-test for means

PHQ-9: Patient Health Questionnaire 9

*Higher maternal depression scores at the study baseline predict lower BMI z-scores at the study endpoint.*

**Fig. 1:**
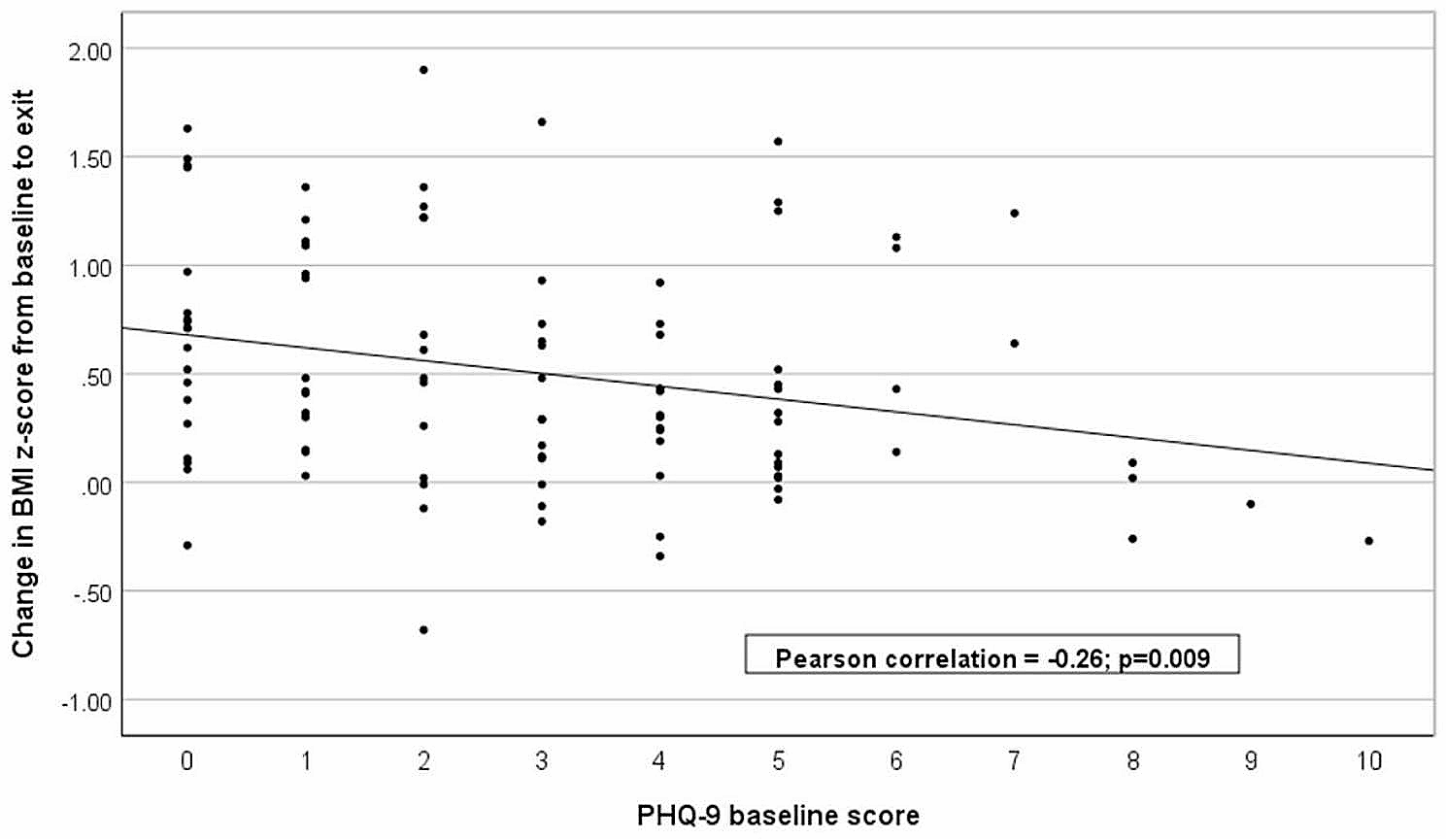
Correlation of the maternal baseline PHQ-9 score and the child’s change in BMI z-score

**Fig. 2:**
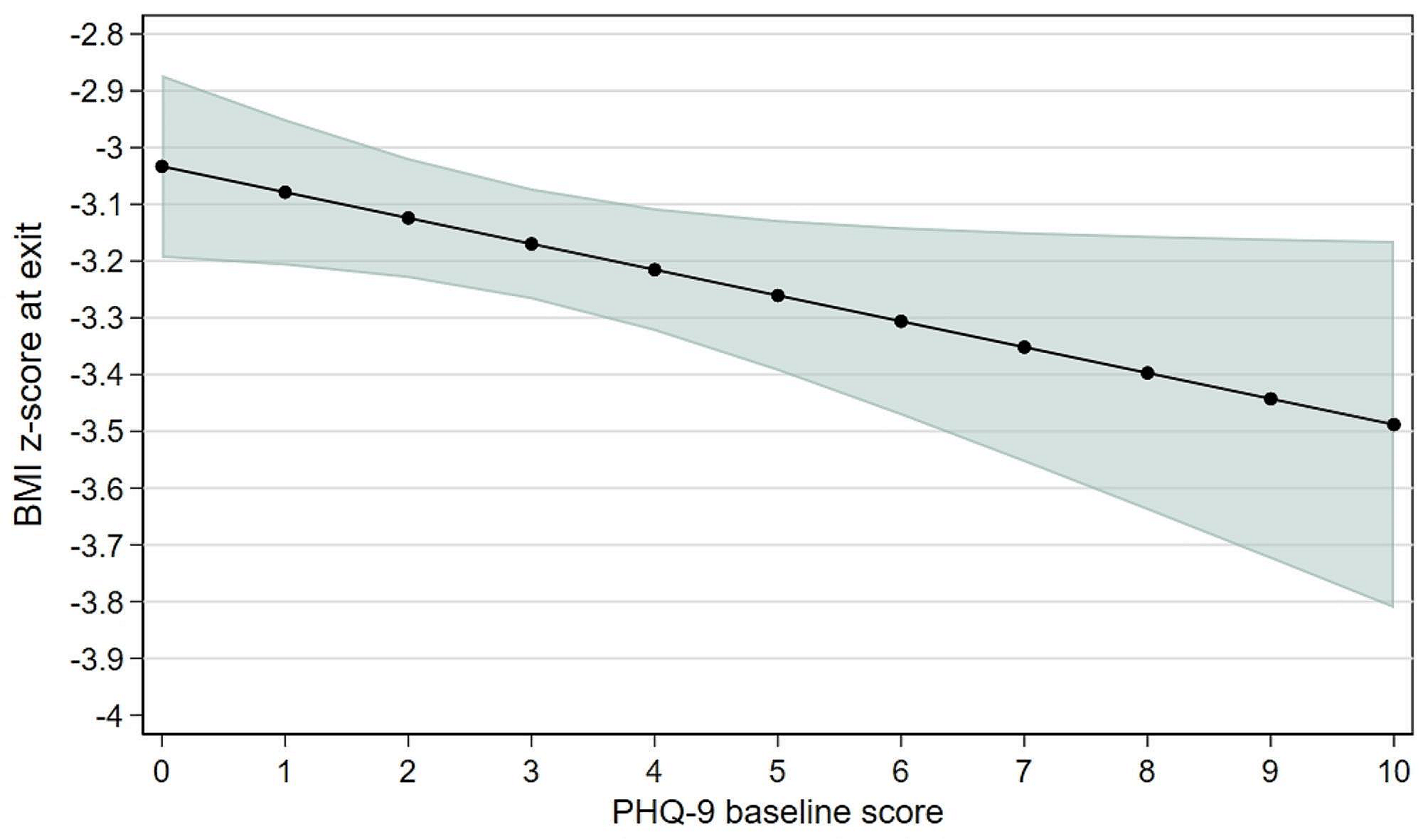
Child’s predicted final BMI z-score with 95% confidence intervals based on maternal baseline PHQ-9 score

Higher levels of baseline maternal depression were associated with a smaller change in BMI z-scores over the 12-week trial period (*r*=-0.26, *p* = 0.009; Fig. 1). We then constructed a multivariable linear regression model for the final BMI z-score, including the maternal baseline PHQ-9 score and the previously identified significant covariates from the SAMS trial (child’s age and baseline BMI z-score) [21]. In the multivariable model, the baseline PHQ-9 score was associated with the final BMI z-score (β=-0.045, *p* = 0.041, Table 3; Fig. 2), and the baseline BMI z-score remained significantly associated with the final BMI z-score (β = 0.666, *p* < 0.001), while age was no longer significantly associated with the final BMI z-score (β=-0.041, *p* = 0.125).

**Table 3:** Multivariable linear regression for the final BMI z-score at the end of the trial

Variables	Beta	95% Confidence Interval	P value
Child Baseline Age	-0.041	-0.093–0.011	0.125
Child Baseline BMI z-score	0.666	0.448–0.884	< 0.001
Maternal Baseline PHQ-9 score	-0.045	-0.089– -0.002	0.041

The dependent variable is the final BMI z-score at the end of the 12-week trial period. The rows report the effect of each baseline covariate in the model by its regression coefficient (Beta), associated 95% confidence interval (CI), and p values

BMI: Body mass index

## Discussion

Maternal depression is an established risk factor for infant-child malnutrition. To our knowledge, for the first time, we demonstrate that the higher a mother’s depressive screening score, the lower her child’s BMI z-score at the end of malnutrition treatment. The baseline maternal depressive symptoms influence the extent of this improvement. Our findings highlight the complex interplay between maternal depressive symptoms and the outcomes of malnutrition treatment in older children with SCA.

Limited studies have explored the effects of maternal mental health on the outcomes of malnutrition treatment in children. For instance, a cross-sectional study conducted seven years after malnutrition treatment failed to establish a significant link between maternal mental health and the current nutritional status of the children [31]. Similarly, another study focused on maternal distress during admission for nutritional rehabilitation found no significant association with child weight gain at a 4-week post-discharge follow-up [32]. Methodological variations, such as differences in follow-up times, could potentially account for the divergent findings in these studies compared to ours. The assessment of maternal distress during inpatient admission may have primarily captured acute distress related to the admission itself because maternal distress levels appear to be consistent regardless of whether the child was admitted for nutritional rehabilitation or other reasons [33]. 

Observational studies linking maternal depression to the development of childhood malnutrition support our finding of the impact of maternal depression on malnutrition treatment outcomes. Children under 5 years old of mothers with depression are more likely to develop malnutrition [10, 13, 34, 35]. In Nigeria, length and weight were lower in the first 6 months of life in children of depressed mothers compared to children of non-depressed mothers [36]. This phenomenon can be at least partially explained by attachment theory, suggesting that a mother’s depressive symptoms may hinder her capacity to respond to her child’s cues, thus affecting both the child’s physical and emotional needs [34, 37, 38]. Maternal depression has also been linked to suboptimal childhood feeding practices [38–40]. Together, these observations underscore the importance of addressing maternal depression as a crucial factor in improving the nutritional status of children.

Limitations of this prospective cohort study, performed as a planned ancillary study to a randomized controlled feasibility trial, include a new onset international conflict that may have limited the food to northern Nigeria, decreasing the response to malnutrition treatment [41–43]. The trial was not designed to test the hypothesis that maternal depression affects the response to malnutrition treatment; thus, our results should be considered hypothesis-generating. We did not evaluate the father’s depression score, which may contribute to the change in the BMI z-score of the trial participant. However, a priori, we had no evidence that the father’s PHQ-9 score would be associated with treatment response during the trial. Additionally, we did not have a measure of maternal education, which impact should be further explored in future studies. Strengths of our study include the high retention rate, detailed data on the children’s response to the interventions, and the innovative approach in examining maternal depressive symptoms as a novel risk factor for suboptimal response to malnutrition treatment.

## Conclusions

In children over 5 years old with SCD in northern Nigeria, a higher screening maternal depression score at the start of malnutrition therapy leads to less improvement in a child’s nutritional status. Based on the results of this study, we have implemented maternal depression screening as standard of care for routine visits in our evaluation of children with uncomplicated severe acute malnutrition. Higher PHQ-9 scores in mothers of children with uncomplicated severe acute malnutrition are further evaluated to determine the mother’s mood and whether she would be amenable to a referral for a formal evaluation.

## Data Availability

The deidentified individual participant data analyzed during this study are available upon request from the corresponding author until 2028, approximately 5 years after publication. After 2028, institutional resources may not be available to provide the data. Requests for data should be directed to lauren.klein@vumc.org, and requestors will need to prepare and sign a data transfer agreement between Vanderbilt University Medical Center and their respective institutions.

## Abbreviations

BMI: Body mass index
DSM-5: Diagnostic and Statistical Manual of Mental Disorders
PHQ-9: Patient Health Questionnaire 9
REDCap: Research Electronic Data Capture
RUTF: Ready-to-use therapeutic food
SCA: Sickle cell anemia
